# Genome Mining Demonstrates the Widespread Occurrence of Gene Clusters Encoding Bacteriocins in Cyanobacteria

**DOI:** 10.1371/journal.pone.0022384

**Published:** 2011-07-20

**Authors:** Hao Wang, David P. Fewer, Kaarina Sivonen

**Affiliations:** Division of Microbiology, Department of Food and Environment Sciences, University of Helsinki, Helsinki, Finland; Instituto de Biología Molecular y Celular de Plantas, Spain

## Abstract

Cyanobacteria are a rich source of natural products with interesting biological activities. Many of these are peptides and the end products of a non-ribosomal pathway. However, several cyanobacterial peptide classes were recently shown to be produced through the proteolytic cleavage and post-translational modification of short precursor peptides. A new class of bacteriocins produced through the proteolytic cleavage and heterocyclization of precursor proteins was recently identified from marine cyanobacteria. Here we show the widespread occurrence of bacteriocin gene clusters in cyanobacteria through comparative analysis of 58 cyanobacterial genomes. A total of 145 bacteriocin gene clusters were discovered through genome mining. These clusters encoded 290 putative bacteriocin precursors. They ranged in length from 28 to 164 amino acids with very little sequence conservation of the core peptide. The gene clusters could be classified into seven groups according to their gene organization and domain composition. This classification is supported by phylogenetic analysis, which further indicated independent evolutionary trajectories of gene clusters in different groups. Our data suggests that cyanobacteria are a prolific source of low-molecular weight post-translationally modified peptides.

## Introduction

Bacteriocins are secondary metabolites and have been found in all major lineages of bacteria [1]. They form a diverse group of small peptides which are often viewed as a part of an elaborate chemical defense system [2]. Bacteriocins are crafted from short ribosomally produced precursor proteins that consist of a C-terminal core peptide and a conserved N-terminal leader sequence, which a processing peptidase recognizes and cleaves [3]. The leader sequence of bacteriocin precursors commonly contains a double glycine motif [3], which is processed by a C39 peptidase domain [4]. The core peptide may undergo further post-translational modifications such as lanthionine formation [5], macrocyclization [5], dehydration [6], or heterocyclization [7], [8]. The proteins involved in the modification, export, and regulation of bacteriocins are often encoded by genes adjacent to the genes encoding the precursor protein [9]–[11]. Many bacteriocins have antimicrobial activity and find applications as food preservatives [12] and antibiotics [13], [14]. During the last decades, bacteriocin research has focused mostly on Gram-positive bacteria, especially lactic acid bacteria [15]. A more detailed structural analysis of bacteriocin gene clusters and precursor peptides in broader families of bacteria most likely will yield invaluable insight into features that are important for their biosynthesis, mode of action, and potential applications.

Cyanobacteria are a prolific source of natural products and secondary metabolites [16], [17]. The biosynthesis of cyanobacterial peptides on non-ribosomal peptide synthetases has been widely demonstrated [18]. Cyanobactins and microviridins were recently shown to be the post-translationally modified peptides in a number of cyanobacteria strains [19]. A genome-wide *in silico* screening for bacteriocins in Gram-negative bacteria had revealed the presence of sixteen double-glycine-type precursors and ten cognate transporters from strains of the cyanobacteria *Nostoc*, *Prochlorococcus, Synechococcus* and *Synechocystis* [20]. The consensus sequence of this double glycine motif was refined to M(R/K)ELX_3_E(I/L)X_2_(I/V)XG(G/A) [20]. A C39 peptidase domain-containing ABC transporter was demonstrated to be a dedicated transporter of double-glycine-type precursors [4]. Two types of such C39 peptidase domain-containing ABC transporters were distinguished in cyanobacteria. The short type composed of an N-terminal C39 peptidase domain, an ABC transporter transmembrane region, and a C-terminal ATP-binding cassette [20]. The long type has an extra 300 amino acids N-terminal extension [20]. Recently, two subclasses of double-glycine-type precursor peptides (NHLP and N11P) were recognized in cyanobacteria [21]. Large scale phylogenetic profiling of bacteria genomes also suggests a link between the biosynthesis of these natural products and a three-gene transport cluster, which includes a C39 peptidase domain-containing ABC transporter, an ABC transporter without peptidase domain, and a secretion protein HlyD [21]. Lantibiotics are a class of extensively modified bacteriocins [5]. A bifunctional lanthionine synthetase (LanM) was discovered from a few cyanobacterial strains and predicted to catalyze macrocyclization and lanthionine formation [22]–[24]. This further guided the identification of lantipeptides from the marine cyanobacterium *Prochlorococcus marinus* MIT9313 [25] and application of incorporating non-proteinogenic residues into natural products [26].

In order to explore the genetic potential for bacteriocin production in cyanobacteria, we mined 58 cyanobacterial genomes to identify the organization of bacteriocin-processing gene clusters. Surprisingly, we found more than a hundred new putative bacteriocin gene clusters from genomes of nearly all examined cyanobacterial species. Nearly 300 putative precursor genes were encoded in close proximity to the bacteriocin gene clusters. Our results demonstrate the widespread presence of bacteriocin gene clusters in cyanobacteria. The genetic diversity of the core peptides of these bacteriocin precursors is enormous with little sequence conservation.

## Results

### Putative cyanobacterial bacteriocin gene clusters and their classification

**Figure 1:**
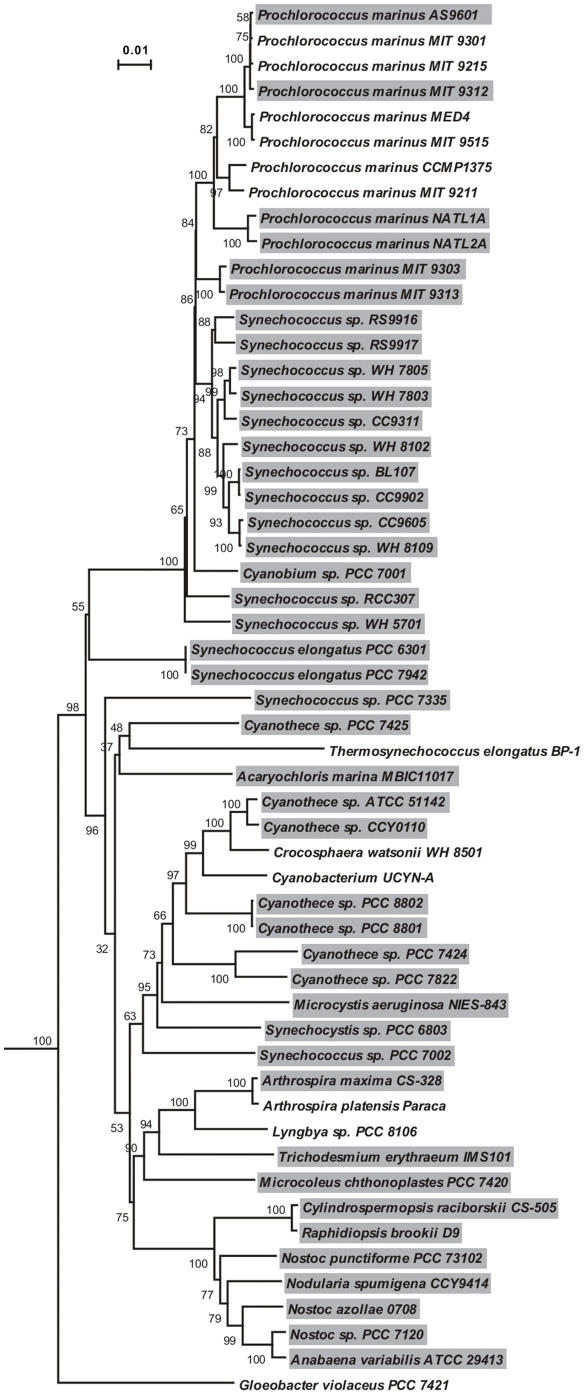
The widespread distribution of putative bacteriocin gene clusters in cyanobacteria. The neighbor-joining tree is based on concatenated 16S and 23S rRNA genes from 55 cyanobacterial genomes. The strains which have at least one bacteriocin gene cluster are indicated with a gray background. Phylogenetic analyses were conducted in MEGA4 [41] by using the Maximum Composite Likelihood model [42] and with 50000 bootstrap replications for each branch. The bootstrap values are shown next to the branches. Outgroup taxa *Gammaproteobacterium* HdN1, *Bradyrhizobium japonicum* USDA 110, and *Escherichia coli* UMN026 were used to root the tree, which is drawn to scale. Strains *Arthrospira* PCC 8005, *Leptolyngbya valderiana* BDU 20041, and *Prochlorococcus marinus* MIT 9202 are absent from this tree because they are partial genomes and have no complete rRNA genes.

**Figure 2:**
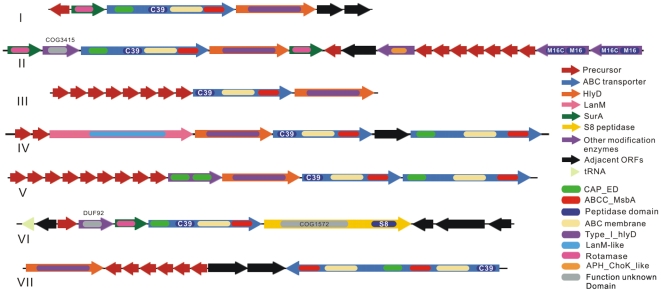
Organization of cyanobacterial bacteriocin gene clusters. The putatively identified gene clusters in this study were classified into seven groups (from I to VII) based on their gene organization and domain composition. ORF sizes and directions are shown in relative scale with color definition as precursor in red, ABC transporter in blue, HlyD in orange, SurA in green, LanM in pink, S8 peptidase-containing protein in yellow, other modification enzymes in purple, adjacent ORFs in black, and tRNA gene in light green. Domains involved in cyanobacterial bacteriocin production and modification are demonstrated within the ORFs with different colors, domain names are derived from the Conserved Domain Database [35]. (I) An example structure of group I from strain *Synechococcus* PCC 7335, and the locus_tag of hlyD is S7335_4080. (II) from *Anabaena variabilis* ATCC 29413, and the locus_tag of hlyD is Ava_4382. (III) from *Nostoc* sp. 7120, and the locus_tag of hlyD is alr5148. (IV) from *Nostoc punctiforme* PCC 73102, and the locus_tag of hlyD is Npun_F5048. (V) from *Nostoc punctiforme* PCC 73102, and the locus_tag of hlyD is Npun_R1804. (VI) from *Anabaena variabilis* ATCC 29413, and the locus_tag of the S8 peptidase domain-containing protein is Ava_4226. (VII) from *Trichodesmium erythraeum* IMS101, and the locus_tag of hlyD is Tery_0894.

A total of 145 putative bacteriocin gene clusters were identified in 43 cyanobacteria (Figure 1, Table 1), by analyzing 58 complete and partial genomes from strains with diverse genomic structures and various morphologies (Table S1). These gene clusters were classified into seven groups by comparison of their diverse gene organization and domain composition (Figure 2).

**Table 1:** Putative bacteriocin gene clusters in cyanobacteria.

Strain	Genome	Groups of bacteriocin gene clusters								Total
	Size (Mb)	I	II	III	IV	V	VI	VII	unclassified	
*Prochlorococcus marinus* MIT 9303	2.7	2	0	0	1	1	0	0	0	4
*Prochlorococcus marinus* MIT 9312	1.71	1	0	0	0	0	0	0	0	1
*Prochlorococcus marinus* MIT 9313	2.41	2	0	0	1	2	0	0	0	5
*Prochlorococcus marinus* NATL1A	1.9	1	0	0	0	0	0	0	0	1
*Prochlorococcus marinus* NATL2A	1.8	1	0	0	0	0	0	0	0	1
*Prochlorococcus marinus* AS9601	1.7	1	0	0	0	0	0	0	0	1
*Cyanobium* PCC 7001		1	0	2	0	0	0	0	1	4
*Synechococcus* PCC 7335		1	0	0	0	1	0	0	0	2
*Synechococcus* RS9916		1	0	0	1	1	0	0	0	3
*Synechococcus* BL107		1	0	0	0	0	0	0	1	2
*Synechococcus* WH 7805		2	0	0	0	0	0	0	1	3
*Synechococcus* WH 5701		2	0	0	0	0	0	0	0	2
*Synechococcus* RS9917		1	0	0	0	0	0	0	0	1
*Synechococcus* sp WH 8102	2.43	3	0	0	0	0	0	0	1	4
*Synechococcus* RCC307	2.2	1	0	0	0	0	0	0	0	1
*Synechococcus* CC9902	2.2	2	0	0	0	0	0	0	0	2
*Synechococcus elongatus* PCC 6301	2.7	1	0	1	0	0	0	0	0	2
*Synechococcus elongatus* PCC 7942	2.75	1	0	1	0	0	0	0	0	2
*Synechococcus* PCC 7002	3.4	1	0	0	0	0	0	0	0	1
*Synechococcus* WH 7803	2.4	2	0	0	0	1	0	0	0	3
*Synechococcus* CC9311	2.61	1	0	0	0	0	0	0	1	2
*Synechococcus* CC9605	2.51	1	0	0	0	1	0	0	0	2
*Synechococcus* sp WH 8109		1	0	0	0	0	0	0	0	1
*Cyanothece* PCC 7425	5.82	0	0	0	4	0	0	1	0	5
*Cyanothece* PCC 8802	4.83	1	2	0	1	0	0	0	0	4
*Cyanothece* PCC 7424	6.52	1	2	0	0	0	0	0	0	3
*Cyanothece* PCC 8801	4.81	1	2	0	1	0	0	0	0	4
*Cyanothece* ATCC 51142	5.46	3	1	0	0	0	0	0	0	4
*Cyanothece* PCC 7822		1	0	0	0	0	0	0	0	1
*Cyanothece* CCY0110		1	2	0	0	0	0	0	0	3
*Microcystis aeruginosa* NIES 843	5.8	1	1	0	0	0	0	0	0	2
*Synechocystis* PCC 6803	3.95	1	0	0	0	0	0	0	1	2
*Arthrospira maxima* CS 328		1	0	0	0	0	0	0	0	1
*Microcoleus chthonoplastes* PCC 7420		1	2	0	1	0	0	0	1	5
*Trichodesmium erythraeum* IMS101	7.8	1	1	1	0	0	0	1	0	4
*Acaryochloris marina* MBIC11017	8.36	3	0	1	0	0	0	0	0	4
*Nostoc punctiforme* PCC 73102	9.01	2	2	1	4	3	1	1	0	14
*Nostoc* sp 7120	7.2	3	2	3	1	1	1	0	1	11
*Nostoc azollae* 0708	5.4	1	1	1	0	0	1	0	0	4
*Anabaena variabilis* ATCC 29413	7.07	1	1	2	1	1	1	0	1	8
*Nodularia spumigena* CCY9414		1	3	2	0	0	1	0	0	7
*Cylindrospermopsis raciborskii* CS505		1	1	2	0	0	1	0	0	5
*Raphidiopsis brookii* D9		1	1	2	0	0	0	0	0	4
Total		57	23	19	16	12	6	3	9	145

Group I was the most abundant type with 57 gene clusters and present in one to three copies in all but one cyanobacterial genomes (Table 1). Group II was the second most abundant type, and possess 23 gene clusters found in fifteen genomes. A total of 19 group III bacteriocin gene clusters appeared in twelve strains. Gene clusters encoding LanM proteins were classified as group IV in this study. *Cyanothece* PCC 7425 and *Nostoc punctiforme* PCC 73102 each have four LanM encoding gene clusters, the other strains each have only one. Twelve gene clusters belong to group V were found in nine genomes (Table 1). The gene clusters of group VI were defined as the presence of proteins with S8 peptidase domain, and mostly found from filmentatous diazotrophic strains (Table 1). Group VII had three members found in *Cyanothece* PCC 7425, *Nostoc punctiforme* PCC 73102, and *Trichodesmium erythraeum* IMS101 (Table 1). The majority of these putative gene clusters were encoded on the chromosome. However, eight gene clusters were found in plasmids from five strains. The filamentous heterocyst-forming cyanobacterial strains, which usually have larger genomes, tend to possess more gene clusters than the unicellular marine strains. For example, *Nostoc punctiforme* PCC 73102 had the maximum number of fourteen bacteriocin gene clusters that cover all the seven groups (Table 1). In addition, we found eleven incomplete gene clusters, which have only separate modification or transportation gene/domain and could not be classified into any previously described groups (Table 1). Genomic rearrangements, including truncations, insertions, and frameshifts, were frequently discovered from these putative gene clusters, which can be partially attributed to transposase activities.

Comparative genomic analysis illustrates that conserved domains are arranged in different combinations, and formed the basis of our classification of these gene clusters (Figure 2, Table S2). Two types of C39 peptidase domain-containing ABC transporters were reported from cyanobacteria as cognate transporter of bacteriocins [20]. The short type appears in gene cluster groups III, IV, and V and contains a C39 peptidase, an ABC transmembrane and an ATP-binding cassette domain (Figure 2, Table S2). The long type is found in groups I, II, and VI and differs from the short type in an extra N terminal nucleotide-binding domain (CAP_ED) with putative transcriptional regulative function (Figure 2, Table S2). Interestingly, there are also bimodular proteins consisting of only two CAP_ED domains encoded in group V gene clusters (Figure 2). The gene clusters of groups IV and V encode an additional ABC transporter, which has similar domain composition as the long type transporter but lacks the C39 peptidase domain (Figure 2). All three group VII gene clusters encode a large protein, which appears to be a direct fusion of the short type ABC transporter with C39 peptidase domain and the ABC transporter without the peptidase domain (Figure 2). The type_I_hlyD and rotamase domains are responsible for peptide secretion and modification (Table S2). They are found in single domain proteins HlyD [27] and SurA [28]. HlyD is found in every gene cluster group, while SurA is located in groups I, II, and VI (Figure 2). These six domains are likely to be integral to the biosynthesis of bacteriocins since they are nearly found in every group of cyanobacterial bacteriocin gene clusters (Figure 2, Table S2).

Seven of the LanM encoding gene clusters possess HlyD and the short type transporter. Five of them also have the additional ABC transporter without the peptidase domain (Figure 2). The other nine *lanM* genes are distributed in distant genomic locations and not physically associated with other biosynthetic genes. A total of eight domains, including the rotamase domain, were firstly discovered with putative activity in bacteriocin biosynthesis in this study due to their frequent presence in specific gene cluster groups (Figure 2, Table S2). The presence of M16 and S8 peptidase domains denotes more cleavage sites besides the double glycine motif on the precursors. However, the others are domains with predicted or unknown function (Table S2).

### Diverse precursor proteins located in regions surrounding the putative gene clusters

A total of 290 precursor proteins were predicted from the analyzed cyanobacterial genomes, with size ranging from 28 to 164 aa, by screening the regions surrounding these putative gene clusters (Table 2, Table S3). The genes encoding precursor proteins were found densely arrayed, from one up to sixteen copies, together with other bacteriocin modification enzymes (Figure 2). Most of the precursor peptides we found have not been reported before and were improperly annotated (Table S3). Based upon our gene cluster classification, more than three precursors were found from bacteriocin gene clusters on average in groups II, III, IV, and V (Table 2). A lower number of precursor proteins were encoded in group I and group VI gene clusters.

**Table 2:** Summary of identified bacteriocin precursors and their classification.

Gene cluster groups	Size Range (aa)	NHLP	N11P	HetP	DUF37	Other	Subtotal	Novel *	Average #
I	45–164	1	2	1	1	15	20	1	0.35
II	39–152	12	7	10	0	58	87	11	3.78
III	29–129	26	6	0	2	40	74	21	3.89
IV	31–151	16	29	0	1	15	61	4	3.81
V	28–151	11	10	0	0	16	37	9	3.08
VI	82–84	0	0	0	2	0	2	0	0.33
VII	50–116	0	1	0	0	6	7	0	2.33
Unclassfied	159	0	0	2	0	0	2	0	0.22
Total		66	55	13	6	150	290	46	

*Number of novel precursor genes that are absent from the current genomic annotation.

# Average number of precursor per gene cluster in different groups.

**Figure 3:**
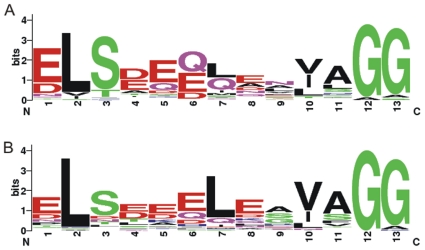
Sequence logos of double-glycine motif generated from cyanobacterial precursors in NHLP and N11P families. There is a conserved region found near the peptide cleavage site with Gly-Gly motif from the precursor peptides. Relative frequency of acidic residues of the conserved sequences from families (A) NHLP and (B) N11P are demonstrated. This figure was generated by web-based software [39].

**Figure 4:**
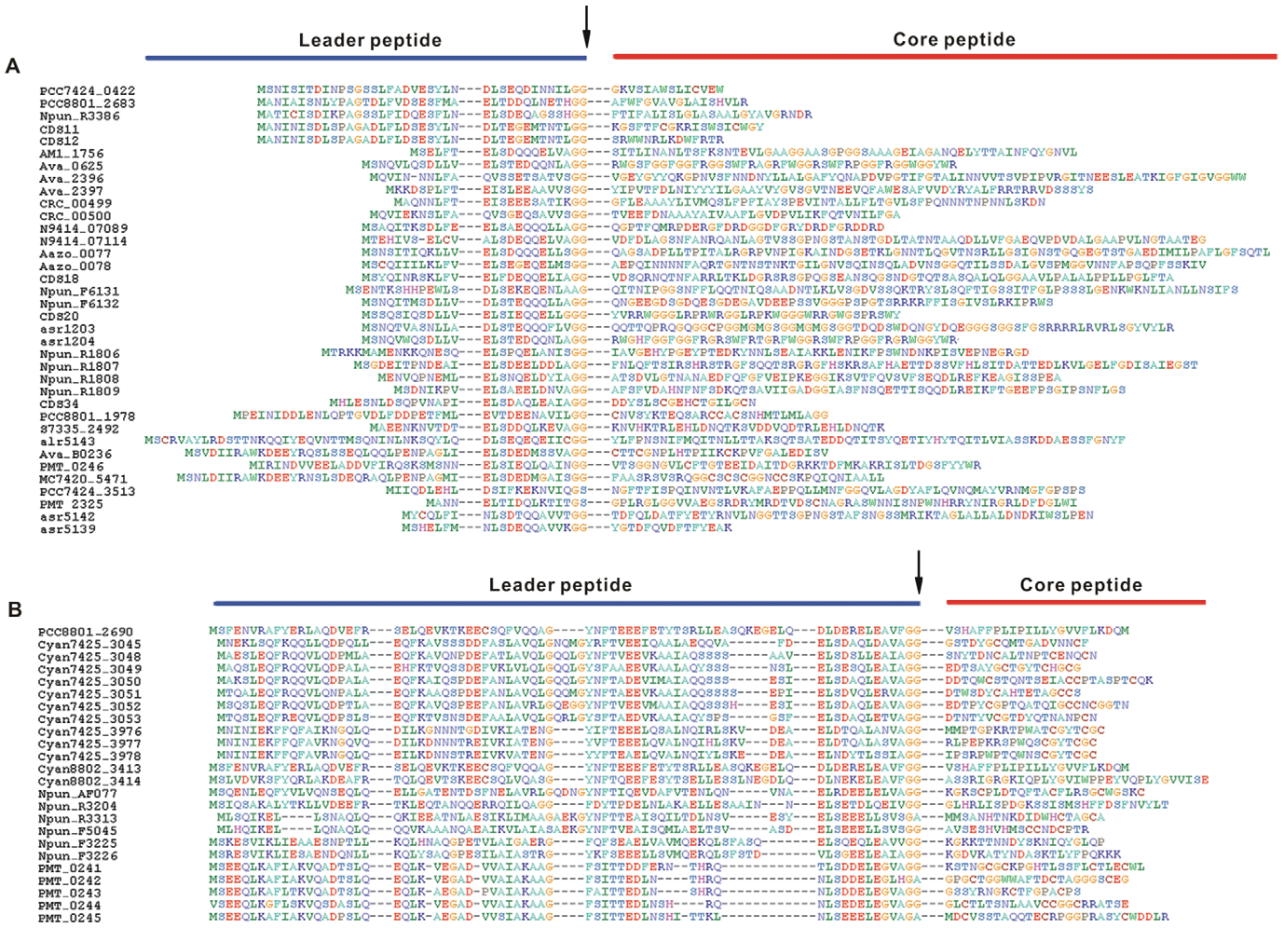
Diverse structures of cyanobacterial bacteriocin precursors from known families. (A) Thirty-nine selected NHLP precursors are shown in a ClustalW alignment [38]. The locus tag is given to the left of the sequence and the amino acid position is given on the right. The cleavage site of the leader peptides is indicated by an arrow. (B) Twenty-four selected N11P precursors shown in a ClustalW alignment. The coloring scheme and notation are identical to section A.

Two protein families of double-glycine-type precursors, NHLP and N11P, were recently redefined [21]. A total of 121 identified precursors can be classified into these two families based on sequence similarity (Table 2). Sequence logos of double glycine motif generated from precursors in the two families display the conservation between them (Figure 3), and to the motif in other bacteria [20], [21]. Sequence alignments of the core peptides reveal a high level diversity both in size and amino acid composition with rich Gly, Cys, Ser and Thr residues that may undergo posttranslational modifications (Figure 4). Moreover, in this study, we also discovered 46 novel precursor proteins which are absent from the current genomic annotation (Table 2); eleven of them were grouped into NHLP and N11P families (Table S3).

HetP proteins are involved in the formation of heterocysts [29], which are specialized nitrogen-fixing cells in cyanobacteria. In this study, HetP-like proteins were found frequently adjacent to cyanobacterial bacteriocin gene clusters and heavily skewed to group II (Table 2). We further discovered a putative cleavage motif KIXDLXYLEX_10_GG from HetP proteins (Figure S1.A, Figure S2), which might be attributed to another peptidase domain M16 found in group II gene clusters. Proteins with DUF37 domain are also found close to bacteriocin gene clusters and possess conserved double glycine (Figure S1.B). HetP and DUF37 family proteins are short and may serve as precursors of bacteriocins (Table S2). Note that precursors in these two families display opposite physical properties to the precursors from NHLP and N11P families, which are mostly in negative charge states (Table S3).

### Phylogenetic analysis of C39 peptidase domain-containing ABC transporters

**Figure 5:**
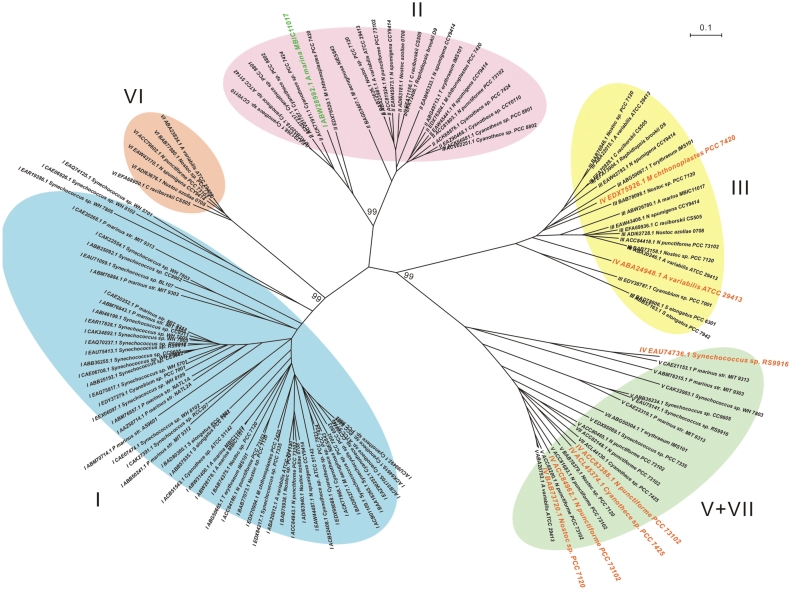
Evolutionary relationships of C39 peptidase domain-containing ABC transporters in cyanobacteria. The phylogenetic analysis is based on C39 peptidase domain-containing ABC transporters in the seven gene cluster groups, except those with disrupted ORFs. The midpoint neighbor-joining tree was constructed by using MEGA4 [41] with Poisson correction model [43] and 50000 bootstrap replications for each branch. The tree is drawn to scale, and bootstrap values of major branches are shown. The name of each taxon is constituted by gene cluster group, accession number, and strain name of the C39 peptidase domain-containing ABC transporters. Major clades of the tree are composed of proteins distinctly from respective gene cluster groups and are named as the corresponding groups with different background colors. Independent evolutionary histories were inferred between the gene clusters in different groups. Proteins from group IV scattered among clades of III and V+VII, and are highlighted in orange. The group I protein found in clade II is shown in green.

A phylogenetic analysis was performed based on the C39 peptidase domain-containing ABC transporters in the seven gene cluster groups (Figure 5). Although the phylogenetic tree was constructed only from the C39 peptidase ABC transporters, it is shown that the branching of this tree matches very well to the grouping based on genetic organization of the gene clusters (Figure 5). Proteins from group V and VII cluster together and corroborates the conclusion that group VII was derived from group V through a recent domain fusion. The partition of clades III and V+VII in the tree is consistent with the absence or presence of the ABC transporter without peptidase domain in the gene cluster groups (Figure 5). The phylogeny shows that group I, II, and VI are closely related. This conforms to the presence of not only long type C39 peptidase domain-containing ABC transporter but also the rotamase domain-containing SurA in their gene clusters (Figure 2). However, clade VI displays a closer relationship to clade I than clade II, though group I gene cluster seems part of group II according to their genetic organization and they would be just same if additional modification genes of group II were removed by genomic rearrangements (Figure 2). This can explain the only inconsistency in the tree, where one group I protein from *Acaryochloris marina* MBIC11017 was found in the clade II (Figure 5). Interestingly, the C39 peptidase domain-containing ABC transporters belong to group IV were not clustered together, but scattered among the clades III and V+VII (Figure 5). We further found that all proteins in clade V+VII come from gene clusters containing the ABC transporter without peptidase domain, and thus possessing the three-gene transport cassette [21].

## Discussion

An initial genome mining study demonstrated the presence of bacteriocin biosynthetic machinery [20]. A recent study demonstrates that cyanobacteria produce bacteriocin-like peptides [25]. Here we show that the genetic machinery for making bacteriocins is widespread in cyanobacteria (Figure 1, Table 1). Previous phylogenetic analysis of C39 peptidase domains demonstrated that cyanobacterial domains clustered together and were separated from those in other Gram-negative and Gram-positive bacteria [20]. Our analysis shows that the C39 peptidase domain-containing ABC transporters from cyanobacteria form different groups that evolve independently (Figure 5).

Lantibiotics form a subclass of bacteriocins due to their specific intramolecular ring structures [30]. The LanM protein was particularly discovered in the genomes of *Nostoc*, *Anabaena*, *Synechococcus*, *Prochlorococcus*, *Cyanothece*, and *Microcoleus* through homolog searches [22]–[24]. In this study, the sixteen LanM-containing gene clusters with diverse genetic organization are classified as group IV. There are nine *lanM* genes found without associated bacteriocin biosynthetic machinery, like the one in *Prochlorococcus marinus* MIT9313 which is the only strain that has been demonstrated to produce bacteriocins in cyanobacteria to date [25]. However, the distribution of C39 peptidase domain-containing ABC transporters from LanM-containing gene clusters suggests a closer relationship to groups III, V, and VII (Figure 5). For example, the stand alone LanM tailoring enzymes in strain *Prochlorococcus marinus* MIT9313 seems to work together with the two group V gene clusters within the same genome for the lantipeptide production [25].

Natural products of ribosomal origin are often derived from the proteolytic cleavage of small precursor proteins, and this strategy appears to be widespread in nature [3]. In cyanobacteria, bacteriocin precursors were recently expanded by identifying two new protein families of double-glycine-type precursors NHLP and N11P [21]. Vast amount of lantibiotics produced by strains of *Prochlorococcus* and *Synechococcus* were predicted in marine system by survey of genes encoding LanM and lantipeptide precursor in metagenomic data [25]. Here we further extended this information by identifying hundreds of new putative precursors via genome mining (Table 2, Table S3). The link between these diverse precursors and the gene clusters with varied domain composition (Figure 2) is the intrinsic specificity to the classic double glycine motif exerted by the C39 peptidase domain [4]. These pieces of evidence would lend support to the conclusion of substrate promiscuity in lantipeptide biosynthesis discovered from marine cyanobacterium *Prochlorococcus marinus* MIT9313 [25]. Consequently, it can be expected that cyanobacteria will become important research subjects of ribosomally synthesized natural products [31].

Research on natural products has been significantly impacted by the surge of genome data in finding new lead structures [32] and discovering new precursors [21]. Bacteriocin finding software and tools often look for precursor genes before locating the gene clusters in screening genomic sequence [33], [34]. However, direct identification of precursor genes from genomic data has been obstructed by their compact sizes and they are often overlooked in conventional annotation. In this study, we firstly identified the putative bacteriocin gene clusters by locating several modification enzymes encoded by conserved genes with large ORFs, which are unlikely to be missed from homolog search. The large amount of precursors identified from this pipeline proved the effectiveness of our method, in spite of the precursors distantly located to the gene clusters [21], [25].

### Conclusion

Cyanobacteria are a prolific source of biologically active peptides with interesting pharmaceutical applications. Here we demonstrate the widespread occurrence of bacteriocin gene clusters in cyanobacteria. These gene clusters can be classified into seven groups according to the diverse organization of catalytic domains within the clusters. Phylogenetic analyses support the gene cluster classification, and show their relatively independent evolutionary histories. Just a small number of these clusters encode the enzymatic machinery necessary to form lanthionines. Hundreds of novel precursors with highly diverse core peptides structures were identified within these gene cluster regions. Although the products of most of the precursor proteins are completely unknown and awaiting verification, it is no doubt that cyanobacteria are emerging as a prolific source of post-translationally modified peptides. The organized information given here would be useful in gaining further information on biosynthetic mechanism of bacteriocins. In addition, this bioinformatic study will not only improve the bacteriocin gene cluster annotation in cyanobacteria but also complement other tools in discovering novel bacteriocins.

## Materials and Methods

### Data sets

Genomic data of 58 cyanobacterial strains (Table S1) were downloaded from the Genbank database (ftp://ftp.ncbi.nih.gov/genbank/). Protein sequences of these genomes were extracted and formatted for local BLAST searches. Three tailored query files containing FASTA format protein sequences of bacteriocin synthesis genes were constructed. The first one consisted of 30 representative proteins of the C39 peptidase domain-containing ABC transporter (cd02259), the second contained 14 HlyD family proteins (TIGR01843). Sequences in these two files were collected from the NCBI Conserved Domains database [35]. The 14 sequences in the third file were LanM proteins located in cyanobacteria [23].

### Gene cluster identification and classification

The three query files were utilized for searching against the database containing all proteins of the collected cyanobacterial genomes. Protein hits of blastP (*E* < 0.00001) [36] were chosen as candidates and labeled in the GenBank format genome sequences, which were used to visualize gene organizations surrounding the candidate proteins by using Artemis [37] for gene cluster identification and intensive structural comparison. The component domains of candidate proteins were identified by Conserved Domain search [35]. Then these putative gene clusters were divided into seven groups by combining the information of gene organization and domain composition.

### Precursor gene identification

Precursor genes were searched in a 20 Kb range upstream and downstream for each gene cluster. Small ORFs and intergenic regions were manually checked by searching for the double glycines. These predicted precursors were compared to known precursor families [21] by blastP (*E* < 0. 1) [36] for classification. Multiple sequence alignments were performed by using ClustalW [38]. Motifs showing the relative frequency of amino acids in leader peptides cleavage region were drawn online by WebLogo [39]. Precursor features were calculated by using Pepstats [40].

### Phylogenetic analysis

Two neighbor-joining trees, both with 50000 bootstrap replications for each branch, were constructed by using the MEGA package (Version 4.0) [41]. The first tree (Figure 1) is based on concatenated 16S and 23S rRNA genes from 55 cyanobacterial genomes with the Maximum Composition Likelihood model [42], and rooted by rRNA genes from *Gammaproteobacterium* HdN1, *Bradyrhizobium japonicum* USDA 110, and *Escherichia coli* UMN026. The second midpoint tree (Figure 5) is generated with Poisson correction model [43] and from amino acid sequences of C39 peptidase domain-containing ABC transporter from all seven gene clusters groups, excluding disrupted ORFs.

## Supporting Information

Figure S1 Sequence alignments of putative novel cyanobacterial bacteriocin precursors. (A) Ten selected HetP substrates are shown in a ClustalW alignment [38]. The locus_tag is given to the left of the sequence and the amino acid position is given on the right. An asterisk implies an invariant residue, while the colon and period show positions that are highly and moderately related, respectively. Bold red text indicates the putative leader peptide cleavage motif. (B) Six selected DUF37 substrates are shown in a ClustalW alignment. The coloring scheme and notation are identical to section A.
(PDF)

Figure S2 Sequence logo of motif with double-glycine found in the putative HetP precursors in cyanobacteria. A conserved region was found near the peptide cleavage site with Gly-Gly motif from the putative HetP precursor proteins in cyanobacteria. Here the sequence logo with relative frequency of acidic residues of the conserved sequences is demonstrated. This figure was generated by web-based software [39].
(PDF)

Table S1 Cyanobacterial genomes analyzed in this study (data collected at May 17, 2010).
(PDF)

Table S2 Conserved domains identified in cyanobacterial bacteriocin gene clusters.
(PDF)

Table S3 Cyanobacterial precursors identified in this study.
(XLS)
