# Supplementary material for: Genome Mining Demonstrates the Widespread Occurrence of Gene Clusters Encoding Bacteriocins in Cyanobacteria
Source: PLoS One. 2011 Jul 20;6(7):e22384. doi: 10.1371/journal.pone.0022384 (PMC3140520; doi:10.1371/journal.pone.0022384)
Supplement: Figure S1 — Sequence alignments of putative novel cyanobacterial bacteriocin precursors. (A) Ten selected HetP substrates are shown in a ClustalW alignment [38]. The locus_tag is given to the left of the sequence and the amino acid position is given on the right. An asterisk implies an invariant residue, while the colon and period show positions that are highly and moderately related, respectively. Bold red text indicates the putative leader peptide cleavage motif. (B) Six selected DUF37 substrates are shown in a ClustalW alignment. The coloring scheme and notation are identical to section A. (PDF) [file pone.0022384.s001.pdf]

A

```
Ava_1098           --MNQNTTG--ITNYNKAINPQQFDKVVEAILAGKYSWACVLMLRFAGYNPMHYIPYRTY 56
alr2818            --MNQNTTG--ITNYNKAINPQQFDKVVEAILAGKYSWACVLMLRFAGYNPMHYIPYRTY 56
Aazo_0724          --MNQDISGK-SSNLEKKINPEQFDQVIEAILAGKYSWACVLMLRFVGYNPLHYIPYRTY 57
AM1_4010           --MNHNLSTHSGTDFDKQITYEQFNQVIEAILMGKYSWACVLMLRFLGYNPLHYIPYRTY 58
PCC8801_3266       --MNRR-FSNESGKTDKVMKEEQFEEIVAAILNGKYSWACVLILKFAGYNPLHYIPYRTY 57
Cyan8802_2855      --MNRR-FSNESGKTDKVMKEEQFEEIVAAILNGKYSWACVLILKFAGYNPLHYIPYRTY 57
PCC7424_3517       --MRQQDFSNRFSKKDQVMTQEQFEKIVEAILAGKYSWACVLILRFAGYNPLHYIPYRTY 58
cce_2677           LTMSNSGYSANS-KQSRTMTEEQFEKIVDAILAGKYSWACVLILQTAGYNPLHYIPYRTY 59
CY0110_11572       --MSQSLYQQKN-QVTKSMTEQQFEEIVDAILAGKYSWACVLILQTAGYNPLHYIPYRTY 57
MC7420_4637        --MTYN-MPTINAKREKTLDPEQFEQIIDAIRQGKYSWACVLLLRFAGHNPQYYIPYRTY 57
                     *          .  : :  :**:::: **  *********:*:  *:** :*******

Ava_1098           NRLLKENSEASKVQQPQH-DNLKNSQVAAVSRSNTNMPSSCLSKIKDLAYLEVVGKQTTE 115
alr2818            NRLLKENSEASKVQQQQH-DNLKNSQVAAVSRSNTNMPSSCLSKIKDLAYLEVVGKQTTE 115
Aazo_0724          NRLLKENSRISRSNTQQN-ESLKLAKPATEKRCDTHLASACLSKIKDIAYLEVGGKQKAE 116
AM1_4010           NRLIKKESLLSYQTSKDK-ASCKDFEPNSLIT-----PSNSPRYMQDLEYLEEVREKSTQ 112
PCC8801_3266       NRLIKDNCLNKPSSQEKN-QKTLNKSSDDNGDEFHRISY--HQKIRDLSYLESVDKKAVK 114
Cyan8802_2855      NRLIKDNCLNKPSSQEKN-QKTLNKSSDDNGDEFHRISY--HQKIRDLSYLESVDKKAVK 114
PCC7424_3517       NRLIKDNCRENSSNQEQN-QQKKPNSDLSSRYYNNSSSYRDNSKIKDLNYLEECNQKEKL 117
cce_2677           NRLIKDNCLKQSHKKEKNEQRNFQKQQLSKDSCQQTTTQTSREKIKDINYLEELTNNTAK 119
CY0110_11572       NRLIKDNRLK---KRDSEQQKTIKPS--PKNSVTKHRTST---KISNLSYVESLDESESN 109
MC7420_4637        NRLRKEHSSQKPQENLQSGDTSGNPGQPTRKSS---------CKIADLNHLEVVDHKTLS 108
                   *** *..         .                           : :: ::*   ..

Ava_1098           IHGG NL----DQWLTEQVHEFQDMYLEPQAISNQDITFKLSDLDFIHN 159
alr2818            IHGG NL----DQWLTEQVHEFQDMYLEPQAISNQDITFKLSDLDFIHN 159
Aazo_0724          VRGS HR----EKKSA-------------------------------- 127
AM1_4010           VTGG TS----NNWLAKN---YLSMWNKKSSNPNL------------- 139
PCC8801_3266       VAGG FR----YFWW--------------------------------- 124
Cyan8802_2855      VAGG FR----YFWW--------------------------------- 124
PCC7424_3517       MAGS CG----EKWRIFS------------------------------ 130
cce_2677           IHGG F---RLQLWQF-------------------------------- 131
CY0110_11572       SLGG LGCNQSSIWSIFFWNH--------------------------- 129
MC7420_4637        MKGG YN---LQGLPAIESEDCIPDSVAPSDDWFSGVSSLFNRLTGLG- 152
                     *.
```

B

```
asr1611               ------------------MKQIFIWLIKGYRMFISPLYPPTCRFRPTCSM 32
Ava_4222              ------------------MKQIFIWLIKGYRMFISPLFPPTCRFQPTCSM 32
N9414_07129           ------------------MKLLFIWLIKGYRMFISPLFPPTCRFQPTCSM 32
Aazo_0083             ------------------MKILLIWIIKGYRLFVSPLFPPTCRFQPTCSM 32
S7335_3409            ----------VLFSRFYEMKLTLLLLVKGYRQLISPLFPPMCRFEPTCSR 40
P9303_21151           MRESNTLSGGIFALLNRAIGSVLLALIGFYRTWLSPLLGPHCRFIPSCSA 50
                                        :   :: ::  **  :***  * *** *:**

asr1611               YAIEAIERFGVFRGG WMAIRRILRCHPFHPGG YDPVPELGEH-CCHH--- 78
Ava_4222              YALEAIERFGVFRGG WMGIRRILRCHPFHPGG YDPVPEVGEH-CCHH--- 78
N9414_07129           YAIEAIERFGIWRGG WMATRRILRCHPFHPGG YDPVPEVKHN-CCDQHLS 81
Aazo_0083             YAIQAIERFGLLRGG WMATLRILRCHPFHPGG YDPVPGLAEKSCCDHH-- 80
S7335_3409            YALDAIDRFGPLQGT WLTAKRFCRCHPLHPGG YDPVPEKKSVL------- 83
P9303_21151           YGLEAIQRHGPWRGG WLTLRRLSRCHPFTPCG CDPVPD------------ 88
                      *.::**:*.*  :*  *:   *: ****: * *  ****

asr1611               DSGNKG-------- 84
Ava_4222              DSGK---------- 82
N9414_07129           DSGKQTTEDHHKGS 95
Aazo_0083             D------------- 81
S7335_3409            --------------
P9303_21151           --------------
```

Figure S1. Sequence alignments of putative novel cyanobacterial bacteriocin precursors. (A) Ten selected HetP substrates are shown in a ClustalW alignment [1]. The locus_tag is given to the left of

the sequence and the amino acid position is given on the right. An asterisk implies an invariant residue, while the colon and period show positions that are highly and moderately related, respectively. Bold red text indicates the putative leader peptide cleavage motif. (B) Six selected DUF37 substrates are shown in a ClustalW alignment. The coloring scheme and notation are identical to section A.

1. Thompson JD, Gibson TJ, Higgins DG. (2002) Multiple sequence alignment using ClustalW and ClustalX. Curr Protoc Bioinformatics Chapter 2: Unit 2.3.
