# Supplementary material for: Genome Mining Demonstrates the Widespread Occurrence of Gene Clusters Encoding Bacteriocins in Cyanobacteria
Source: PLoS One. 2011 Jul 20;6(7):e22384. doi: 10.1371/journal.pone.0022384 (PMC3140520; doi:10.1371/journal.pone.0022384)
Supplement: Figure S2 — Sequence logo of motif with double-glycine found in the putative HetP precursors in cyanobacteria. A conserved region was found near the peptide cleavage site with Gly-Gly motif from the putative HetP precursor proteins in cyanobacteria. Here the sequence logo with relative frequency of acidic residues of the conserved sequences is demonstrated. This figure was generated by web-based software [39]. (PDF) [file pone.0022384.s002.pdf]

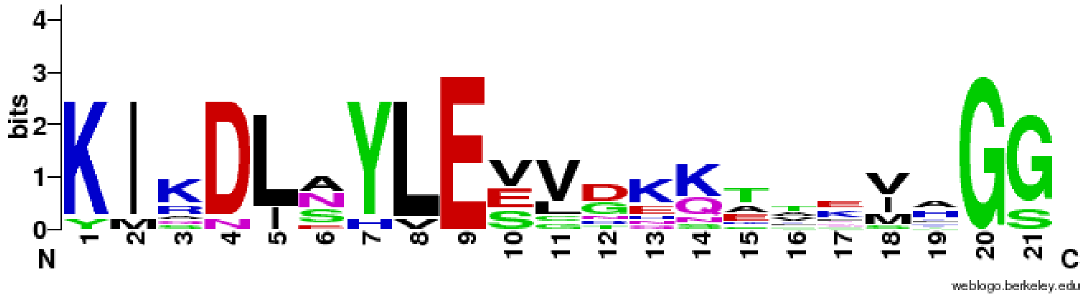

Figure S2. Sequence logo of motif with double-glycine found in the putative HetP precursors in cyanobacteria. A conserved region was found near the peptide cleavage site with Gly-Gly motif from the putative HetP precursor proteins in cyanobacteria. Here the sequence logo with relative frequency of acidic residues of the conserved sequences is demonstrated. This figure was generated by web-based software [1].

1. Crooks GE, Hon G, Chandonia JM, Brenner SE. (2004) WebLogo: A sequence logo generator. Genome Res 14: 1188-1190.
