# Supplementary material for: Genome Mining Demonstrates the Widespread Occurrence of Gene Clusters Encoding Bacteriocins in Cyanobacteria
Source: PLoS One. 2011 Jul 20;6(7):e22384. doi: 10.1371/journal.pone.0022384 (PMC3140520; doi:10.1371/journal.pone.0022384)
Supplement: Table S1 — Cyanobacterial genomes analyzed in this study (data collected at May 17, 2010). (PDF) [file pone.0022384.s003.pdf]

Table S1. Cyanobacterial genomes analyzed in this study (data collected at May 17, 2010)

Strain	Accession Number	Status	Genome Size (Mb)	# Chromsome	# plasmid
Gloeobacter violaceus PCC 7421	BA000045	Complete	4.66	1	0
Prochlorococcus marinus CCMP1375	AE017126	In complete		NA	NA
Prochlorococcus marinus MED4	BX548174	In complete		NA	NA
Prochlorococcus marinus MIT 9202	ACDW00000000	In complete		NA	NA
Prochlorococcus marinus MIT 9211	CP000878	In complete		NA	NA
Prochlorococcus marinus MIT 9215	CP000825	In complete		NA	NA
Prochlorococcus marinus MIT 9301	CP000576	In complete		NA	NA
Prochlorococcus marinus MIT 9303	CP000554	Complete	2.7	1	0
Prochlorococcus marinus MIT 9312	CP000111	Complete	1.71	1	0
Prochlorococcus marinus MIT 9313	BX548175	Complete	2.41	1	0
Prochlorococcus marinus MIT 9515	CP000552	In complete		NA	NA
Prochlorococcus marinus NATL1A	CP000553	Complete	1.9	1	0
Prochlorococcus marinus NATL2A	CP000095	Complete	1.8	1	0
Prochlorococcus marinus AS9601	CP000551	Complete	1.7	1	0
Cyanobium PCC 7001	DS990556	In complete		NA	NA
Synechococcus PCC 7335	DS989904	In complete		NA	NA
Synechococcus RS9916	DS022299	In complete		NA	NA
Synechococcus BL107	DS022298	In complete		NA	NA
Synechococcus WH 7805	CH724168	In complete		NA	NA
Synechococcus WH 5701	AANO01000000	In complete		NA	NA
Synechococcus RS9917	CH724158	In complete		NA	NA
Synechococcus sp WH 8102	BX548020	Complete	2.43	1	0
Synechococcus RCC307	CT978603	Complete	2.2	1	0
Synechococcus CC9902	CP000097	Complete	2.2	1	0
Synechococcus elongatus PCC 6301	AP008231	Complete	2.7	1	0
Synechococcus elongatus PCC 7942	CP000100	Complete	2.75	1	1
Synechococcus PCC 7002	CP000951	Complete	3.4	1	6
Synechococcus WH 7803	CT971583	Complete	2.4	1	0
Synechococcus CC9311	CP000435	Complete	2.61	1	0
Synechococcus CC9605	CP000110	Complete	2.51	1	0
Synechococcus sp WH 8109	GG704594	In complete		NA	NA
Cyanothece PCC 7425	CP001344	Complete	5.82	1	3
Cyanothece PCC 8802	CP001701	Complete	4.83	1	4
Cyanothece PCC 7424	CP001291	Complete	6.52	1	6
Cyanothece PCC 8801	CP001287	Complete	4.81	1	3
Cyanothece ATCC 51142	CP000806	Complete	5.46	2	4
Cyanothece PCC 7822	ABVE00000000	In complete		NA	NA
Cyanothece CCY0110	AAXW01000000	In complete		NA	NA
Microcystis aeruginosa NIES 843	AP009552	Complete	5.8	1	0
cyanobacterium UCYN A	CP001842	In complete		NA	NA
Thermosynechococcus elongatus BP-1	BA000039	Complete	2.59	1	0
Synechocystis PCC 6803	BA000022	Complete	3.95	1	4

Crocosphaera watsonii WH 8501	AADV00000000	In complete		NA	NA
Arthrospira maxima CS 328	ABYK01000000	In complete		NA	NA
Arthrospira PCC 8005	ADDH00000000	In complete		NA	NA
Arthrospira platensis str. Paraca	ACSK00000000	In complete		NA	NA
Microcoleus chthonoplastes PCC 7420	ABRS01000000	In complete		NA	NA
Trichodesmium erythraeum IMS101	CP000393	Complete	7.8	1	0
Leptolyngbya valderiana BDU 20041	AAZV00000000	In complete		NA	NA
Lyngbya PCC 8106	AAVU00000000	In complete		NA	NA
Acaryochloris marina MBIC11017	CP000828	Complete	8.36	1	9
Nostoc punctiforme PCC 73102	CP001037	Complete	9.01	1	5
Nostoc sp 7120	BA000019	Complete	7.2	1	6
Nostoc azollae 0708	CP002059	Complete		NA	NA
Anabaena variabilis ATCC 29413	CP000117	Complete	7.07	1	3
Nodularia spumigena CCY9414	AAVW01000000	In complete		NA	NA
Cylindrospermopsis raciborskii CS 505	ACYA01000000	In complete		NA	NA
Raphidiopsis brookii D9	ACYB01000000	In complete		NA	NA

NA - not applicable
