# Supplementary material for: Genome Mining Demonstrates the Widespread Occurrence of Gene Clusters Encoding Bacteriocins in Cyanobacteria
Source: PLoS One. 2011 Jul 20;6(7):e22384. doi: 10.1371/journal.pone.0022384 (PMC3140520; doi:10.1371/journal.pone.0022384)
Supplement: Table S2 — Conserved domains identified in cyanobacterial bacteriocin gene clusters. (PDF) [file pone.0022384.s004.pdf]

Table S2. Conserved domains identified in cyanobacterial bacteriocin gene clusters.

Domain Name	Domain Description	Presence in Groups
C39 peptidase	Bacteriocin-processing C39 family peptidase	I, II, III, IV, V, VI, VII
CAP_ED	Effector domain of the CAP family of transcription	I, II, IV, V, VI, VII
ABC membrane	Transmembrane domain with six helices	I, II, III, IV, V, VI, VII
ABCC_MsbA	ABC transporter domain	I, II, III, IV, V, VI, VII
Type_I_hlyD	Domain found in type I secretion ABC transporter	I, II, III, IV, V, VI, VII
Rotamase *	Increase the rate of protein folding by catalysing the interconversion of cis- and trans-proline	I, II, V, VI
LanM-like	Lanthionine synthetases for dehydration and the cyclization of the precursor-peptide	IV
S8 peptidase *	Serine endo- and exo-peptidase contain an Asp/His/Ser catalytic triad similar to that of trypsin	VI
M16 peptidase *	Insulinase	II
APH_ChoK_like *	Phosphorylation of the antibiotics, aminoglycosides and macrolides.	II
NADB *	NAD(P)H/NAD(P)(+) binding domain found in dehydrogenases	VI
COG3415 *	Transposase and inactivated derivatives	II
DUF92 *	Unknown function domain with predicted transmembrane helices	VI
COG1572 *	Unknown function	VI
HetP #	Unknown function	I, II
DUF37 #	Unknown function domain contains three conserved cysteine residues	I, III, IV, VI

* - Domains may be involved in cyanobacterial bacteriocin biosynthesis found in this study. # - Putative domains of bacteriocin precursors. Domain names are derived from the Conserved Domain Database [1].

1. Marchler-Bauer A, Lu S, Anderson JB, Chitsaz F, Derbyshire MK, et al. (2011) CDD: A conserved domain database for the functional annotation of proteins. Nucleic Acids Res 39: D225-9.
